# Genetic Variants, Lifestyle Factors and Changes in Body Fat Percentage in Young Adults: An Exploratory Prospective Study

**DOI:** 10.3390/nu18213679

**Published:** 2026-11-06

**Authors:** Emiliya S. Egorova, Elena V. Valeeva, Ildus I. Ahmetov

**Affiliations:** 1 Laboratory of Genetics of Aging and Longevity, Kazan State Medical University, Kazan 420012, Russia; 2 Research Institute for Sport and Exercise Sciences, Liverpool John Moores University, Liverpool L3 5AF, UK

**Keywords:** body fat percentage, longitudinal change, genetic polymorphisms, sleep quality, dietary habits, inter-individual variability, young adults, prospective study

## Abstract

**Background/Objectives:** We examined whether eight obesity-associated polymorphisms and four lifestyle exposures were associated with changes in body fat percentage (BF%) in young adults. **Methods:** Eighty-four young adults of European ancestry (82% women; age, 20.5 ± 2.6 years) from Kazan, Russia, underwent bioelectrical impedance analysis at baseline and follow-up, 1.9 ± 0.8 years apart. A subsample was genotyped for eight obesity-associated polymorphisms (*ADCY3* rs11676272, *RERE* rs301806, *FTO* rs1421085, *TNRC6B* rs4821940, *PATJ* rs12140153, *TFAP2B* rs734597, *MMS22L* rs9385223, and *SLC22A3* rs9364554). Dietary intake and sleep characteristics were assessed using questionnaires, including the Pittsburgh Sleep Quality Index (PSQI). Follow-up BF% was analyzed using ANCOVA adjusted for baseline BF%, follow-up duration, age and sex, with false discovery rate (FDR) correction for multiple testing. **Results:** Mean BF% increased by 1.61 ± 3.97 percentage points (pp) in men (*p* = 0.14) and by 0.25 ± 4.43 pp in women (*p* = 0.65), with wide inter-individual variation. Compared with participants in the bottom quartile (BF% change ≤ −2.45 pp), those in the top quartile (BF% gain ≥ 3.38 pp), i.e., those who gained more body fat, were more likely to carry the *FTO* rs1421085 T allele (*p* = 0.02; FDR *q* = 0.157), consumed fewer vegetables (*p* = 0.004; FDR *q* = 0.036), and had longer sleep duration (*p* = 0.028; FDR *q* = 0.126). Furthermore, poorer subjective sleep quality was associated with higher follow-up BF% in the whole cohort (*β* = 1.96 pp per PSQI point; 95% CI, 0.23–3.68; *p* = 0.027; FDR *q* = 0.11). **Conclusions:** Given the modest sample size and relatively short follow-up, few associations were detected, most of which did not remain significant after correction for multiple comparisons. Nevertheless, vegetable intake and sleep characteristics may be relevant to changes in adiposity during young adulthood.

## 1. Introduction

Obesity results from the interplay between genetic susceptibility and modifiable behavioural and environmental factors. Young adulthood is a period of substantial change in dietary habits, eating behaviour, sleep and other lifestyle characteristics, during which adiposity often increases [1]. Body fat percentage (BF%) is a more direct measure of body composition than body mass index (BMI) and may better capture inter-individual differences in adiposity.

Genome-wide association studies (GWASs) have identified hundreds of loci associated with BMI and BF%, implicating pathways involved in appetite regulation, energy homeostasis and adipocyte biology [2,3]. Longitudinal analyses suggest that the genetic architecture underlying changes in adiposity is only partly shared with that underlying adiposity levels [4]. Thus, variants associated with adiposity at a given time point may not necessarily predict subsequent changes in adiposity. Previous longitudinal studies of genetic variants and body composition have predominantly followed populations of European ancestry from childhood or adolescence into adulthood over extended periods [5,6,7], whereas short-term changes during young adulthood and populations from other regions have received considerably less attention.

Lifestyle factors, including diet quality, eating behaviour and sleep, are also associated with obesity risk in young adults, and longitudinal studies have linked poorer sleep with subsequent gains in adiposity [1,8]. In a previous cross-sectional study of students, we observed associations of genetic and lifestyle factors with obesity-related phenotypes [9]. However, whether these factors are associated with subsequent changes in body composition in this population remains unclear.

To our knowledge, no prospective study has examined these variants in relation to changes in measured body composition among young adults in Russia. Moreover, most longitudinal studies report mean changes in adiposity, while relatively little is known about inter-individual variability in body-fat trajectories during young adulthood, including the proportion of individuals who gain or lose substantial amounts of body fat over a relatively short period. Because change scores are inherently related to baseline values, partly as a consequence of regression to the mean, analyses of longitudinal change should account for baseline adiposity [10,11,12].

This exploratory prospective study followed young adults for approximately 1.8 years and had three aims: (i) to estimate the associations, with corresponding measures of precision, between eight polymorphisms previously associated with adiposity and change in BF%, and to provide summary statistics for future studies; (ii) to examine the associations of vegetable intake, fermented dairy intake and sleep quality with change in BF%; and (iii) to characterise inter-individual variability in BF% change.

## 2. Materials and Methods

### 2.1. Study Design and Participants

This prospective cohort study was conducted in accordance with the Declaration of Helsinki and approved by the Local Ethics Committee of Kazan State Medical University (protocol No. 10, 21 November 2023). All participants provided written informed consent.

Participants were students who underwent assessments at the Laboratory of Genetics of Aging and Longevity, Kazan State Medical University. Exclusion criteria were participation in professional sports, pregnancy or lactation, severe chronic disease (including cancer, endocrine disorders and psychiatric conditions), and physical disability preventing routine physical activity. The analysis included 84 participants (69 women and 15 men) with two body-composition measurements, of whom 66 were genotyped for eight DNA polymorphisms. Age at baseline was 20.5 ± 2.6 years (range, 18–27 years).

The baseline examination was conducted as part of a previously reported cross-sectional study of students at Kazan State Medical University [9]. That study examined associations of genetic and lifestyle factors with obesity-related phenotypes at a single time point. The present analysis was restricted to the 84 participants who underwent a second body-composition assessment. The follow-up measurements and all analyses of changes in body fat percentage are reported here for the first time.

Each participant underwent two examinations using the same set of measurements. The follow-up interval was calculated from the dates of the two examinations and was 1.9 ± 0.8 years. No dietary or exercise intervention was prescribed during the follow-up period.

### 2.2. Body Composition

Body composition was assessed by multifrequency bioelectrical impedance analysis (BIA) using the same Tanita MC-780 MA analyzer (Tanita Corporation, Tokyo, Japan) at both examinations. The device was maintained and checked according to the manufacturer’s instructions. Measurements were performed in the morning (08:00–10:00) after an overnight fast, with participants barefoot, wearing light clothing and after voiding their bladder. Participants were instructed to avoid vigorous physical activity for 24 h and alcohol for 48 h before the examination and to refrain from consuming caffeine-containing beverages on the morning of the measurement. Menstrual-cycle phase was not recorded. The primary outcome was body fat percentage (BF%). Change in BF% was calculated as follow-up BF% minus baseline BF% and is expressed in percentage points (pp).

### 2.3. Genotyping

DNA was extracted from buccal epithelial swabs using the DNA-sorb-AM kit (Central Research Institute of Epidemiology, Moscow, Russia). Single-nucleotide polymorphisms (SNPs) were genotyped by real-time PCR on a CFX96 Touch thermal cycler (Bio-Rad, Hercules, CA, USA) using Testgen reagent kits (TestGene LLC, Ulyanovsk, Russia). Eight SNPs were analysed: *ADCY3* rs11676272 (risk allele G), *RERE* rs301806 (T), *FTO* rs1421085 (C), *TNRC6B* rs4821940 (T), *PATJ* rs12140153 (G), *TFAP2B* rs734597 (A), *MMS22L* rs9385223 (G) and *SLC22A3* rs9364554 (T). Negative controls were included in each run.

The SNPs were selected a priori based on genome-wide association studies of body mass index and body fat percentage [2,3]. The risk allele was defined as the allele previously associated with higher adiposity. Because of limited availability of reagent kits, not all samples could be genotyped for every SNP. The Hardy–Weinberg equilibrium was assessed using an exact test [13].

### 2.4. Lifestyle Exposures

Vegetable and fermented dairy intake were each assessed at the first examination using a single frequency item from a structured questionnaire developed by the authors (“Do you eat the following foods?”). Each item had six response categories, ranging from 1 (never) to 6 (daily), with higher scores indicating more frequent consumption. The exact wording and response categories are provided in Supplementary Materials.

Sleep quality during the previous month was assessed using the Pittsburgh Sleep Quality Index (PSQI) [14] using the Russian translation by E.A. Semenova and K.V. Danilenko (2009), which has previously been used in Russian university students [15]. The prespecified sleep exposure was the PSQI global score (range, 0–21), with higher scores indicating poorer sleep quality. Component 1 (subjective sleep quality; 0 = very good to 3 = very bad) was analyzed as a secondary exploratory exposure; the remaining six components are reported in Supplementary Table S6.

### 2.5. Statistical Analysis

The primary analysis used an analysis of covariance (ANCOVA) model in which follow-up BF% was regressed on the exposure of interest, adjusting for baseline BF%, follow-up duration, age and sex. Genotypes were coded additively according to the number of risk alleles (0, 1 or 2). For each exposure, we report the regression coefficient (*β*, expressed as percentage points of BF% per risk allele or per unit increase in the exposure), its 95% confidence interval (CI), the *p* value for the exposure coefficient, and its partial *R*^2^, representing the proportion of residual variance explained by the exposure beyond the covariates. Genotypes were also analyzed as categorical variables; homozygous groups containing fewer than five participants were combined with the corresponding heterozygous group. Model assumptions were assessed using tests of homogeneity of regression slopes, the Shapiro–Wilk test of residual normality and Levene’s test for homogeneity of variance.

Multiple testing was controlled using the Benjamini–Hochberg false discovery rate (FDR) procedure [16]. For the primary model, we additionally calculated heteroscedasticity-consistent (HC3) standard errors [17], percentile bootstrap 95% CIs based on 5000 resamples, and Freedman–Lane permutation *p* values based on 5000 permutations [18]. Family-wise error across the eight SNPs was controlled using a max-T permutation procedure, with genotype rows permuted jointly [19].

Because baseline-adjusted and unadjusted analyses of change address different statistical questions [11,12], the following sensitivity analyses were performed: (i) analysis of change in BF% without adjustment for baseline BF%; (ii) a random-intercept linear mixed-effects model (LMM) fitted by restricted maximum likelihood, including both measurements, time in years and a time × exposure interaction and adjusting for age, sex and their interactions with time [20]; (iii) analysis of annualized BF% change (change divided by follow-up duration) among participants followed for ≥0.5 years; (iv) the primary model restricted to women and to participants followed for ≥0.5 years; and (v) a mutually adjusted model including all lifestyle exposures. Changes in BF% within genotype groups were assessed using paired t-tests and compared between genotype groups using one-way ANOVA and the Kruskal–Wallis test [21]. Estimates (*β*) and standard errors from all genetic analyses are reported as summary statistics (Supplementary Table S1).

Inter-individual variability was characterised using the distribution of BF% change, the proportions of participants who gained or lost at least 2 and 5 pp, and the correlation between baseline and follow-up BF%. Participants with and without genotype data were compared using Welch’s t-test and Fisher’s exact test. The minimum detectable partial *R*^2^ at 80% power was calculated from the non-central F distribution based on the available sample size [22].

In an exploratory analysis, the upper and lower quartiles of BF% change were compared using the Mann–Whitney test and, to account for baseline differences and regression to the mean [10], Firth penalized logistic regression adjusted for baseline BF%, age, sex and follow-up duration.

All analyses were performed in Python 3.11 using NumPy, pandas and SciPy. All statistical tests were two-sided, and *p* < 0.05 was considered nominally statistically significant.

## 3. Results

### 3.1. Participants and Change in Body Fat Percentage

Characteristics of the 84 participants are shown in Table 1. Mean BF% increased by 0.49 ± 4.36 pp over follow-up (*p* = 0.31), by 1.61 ± 3.97 pp in men (*p* = 0.14) and by 0.25 ± 4.43 pp in women (*p* = 0.65), with substantial individual variation (range, −10.1 to +11.1 pp). Changes were heterogeneous: 34 participants (40%) gained ≥ 2 pp, including 12 who gained ≥ 5 pp; 25 (30%) lost ≥ 2 pp, including 9 who lost ≥ 5 pp; and 25 (30%) changed by less than 2 pp. The interquartile range of BF% change was −2.5 to +3.3 pp. Baseline and follow-up BF% were strongly correlated (*r* = 0.80), whereas baseline BF% was inversely correlated with BF% change (*r* = −0.40, *p* < 0.001), a pattern that may partly reflect regression to the mean. Follow-up duration was not correlated with BF% change (Spearman *ρ* = 0.04, *p* = 0.72).

Genotyped participants (*n* = 66) did not differ from non-genotyped participants (*n* = 18) in age, sex, or baseline BF% (22.4% in both groups), but BF% increased more during follow-up in the genotyped group (+1.35 vs. −2.68 pp, *p* = 0.001). Forty of 80 participants (50%) had a PSQI global score > 5. Vegetable and fermented dairy intake were reported in the two highest frequency categories by 88% and 76% of participants, respectively.

### 3.2. Genotype Distributions

Genotype counts and allele frequencies are shown in Table 2. All genotype distributions were consistent with the Hardy–Weinberg equilibrium (exact *p* ≥ 0.20). No *MMS22L* GG homozygotes were observed.

### 3.3. Genetic Variants and Body Fat Percentage

**Figure 1:**
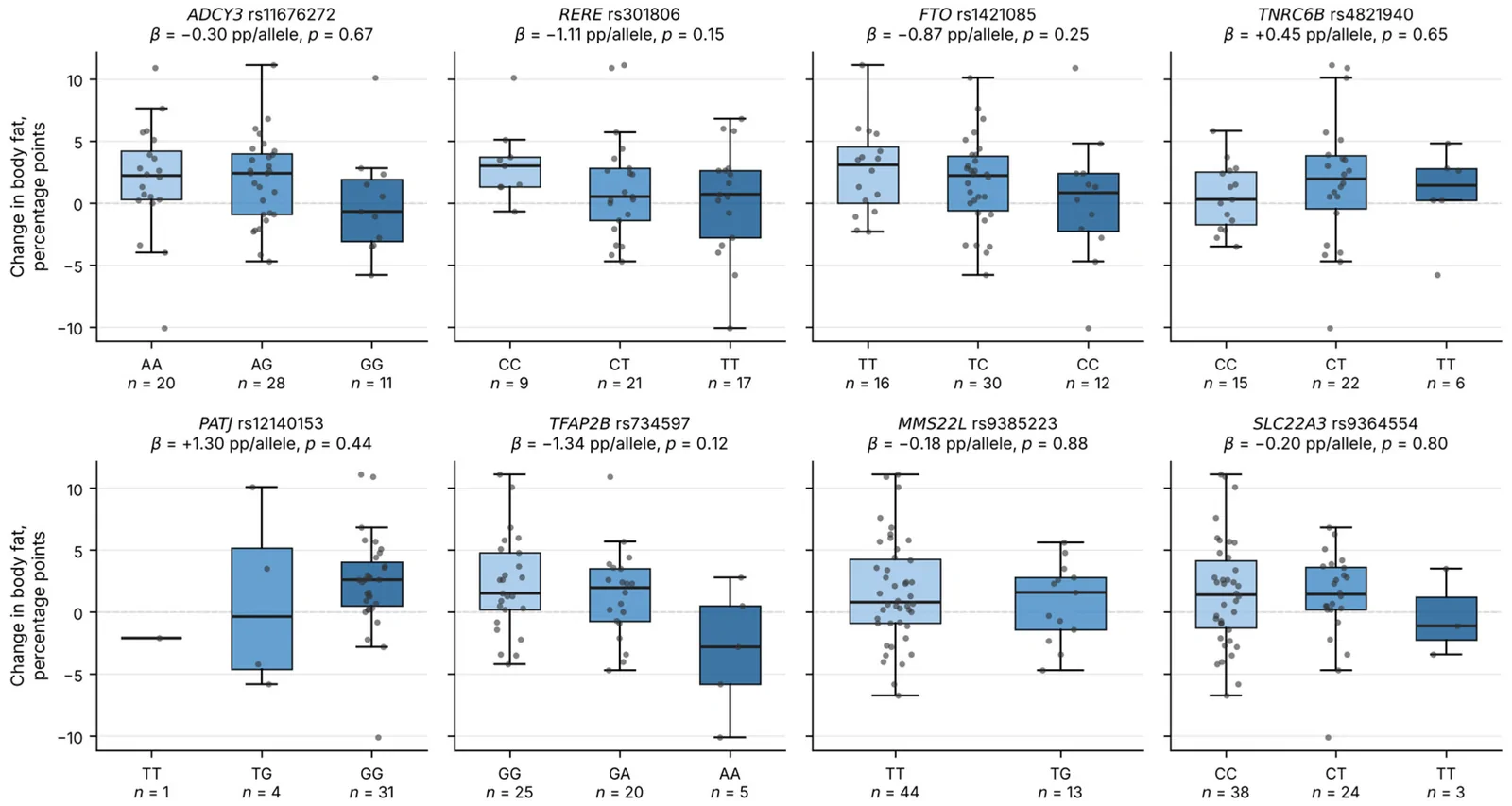
Change in body fat percentage (follow-up minus baseline, percentage points) by genotype for the eight polymorphisms. Boxes show the median and interquartile range, whiskers extend to 1.5 × interquartile range, and points are individual participants; the number of participants is shown under each genotype. Shading becomes darker with increasing numbers of risk alleles. *β* and *p* values in each panel are from the primary ANCOVA model using additive genotype coding and adjusted for baseline BF%, follow-up duration, age, and sex.

No SNP was associated with baseline BF% (all *p* ≥ 0.14) or with follow-up BF% in the primary model (all *p* ≥ 0.12, *q* ≥ 0.59; Table 3, Figure 1). Partial *R*^2^ values for individual SNPs were ≤ 0.054. Results were similar when robust standard errors, bootstrap confidence intervals, and permutation tests were used (all permutation *p* ≥ 0.12; max-T *p* ≥ 0.63); when genotype was analyzed categorically (all *p* ≥ 0.09; Supplementary Table S2); and in subgroup analyses (Supplementary Table S5). The assumption of homogeneous regression slopes was violated for *TNRC6B* (*p* = 0.016). The upper 95% confidence limits indicate that the data are incompatible with per-allele increases in follow-up BF% greater than 0.37 pp (*TFAP2B*) to 2.42 pp (*TNRC6B*) for seven variants, and 4.71 pp for *PATJ* (Table 3). Estimates and standard errors for all models are given in Supplementary Table S1.

In analyses without adjustment for baseline BF%, the *TFAP2B* A allele was nominally associated with a smaller increase in BF% (unadjusted change: *β* = −1.89 pp, *p* = 0.038, *q* = 0.31; LMM time × genotype: *β* = −1.04 pp/year, *p* = 0.030, *q* = 0.24; Supplementary Tables S1 and S3). The same allele was associated with a non-significantly higher baseline BF% (*β* = 2.03 pp, *p* = 0.15), and the association was no longer present after adjustment for baseline BF%. No other SNP was nominally associated with BF% change in any model.

Within individual genotype groups, BF% increased significantly in 22 of 77 paired comparisons across the whole cohort and subgroups, predominantly in the largest groups and irrespective of risk-allele status; none remained significant after FDR correction (*q* ≥ 0.08). This pattern was consistent with the overall increase observed in the genotyped subsample and with differences in group size. Changes did not differ between genotype groups for any SNP (all *q* ≥ 0.37; Supplementary Table S2).

With 36–65 genotyped participants, the study had 80% power at *α* = 0.05 to detect SNP effects explaining ≥ 11–19% of the residual variance, corresponding to effects of ≥1.9–4.7 pp per risk allele.

### 3.4. Lifestyle Exposures and Body Fat Percentage

Vegetable intake, fermented dairy intake, and PSQI global score were not associated with follow-up BF% in the primary model (all *p* ≥ 0.21; Table 3; Supplementary Table S4).

Worse subjective sleep quality was nominally associated with higher follow-up BF% (*β* = 1.96 pp per score point; 95% CI, 0.23–3.68; *p* = 0.027; *q* = 0.11; partial *R*^2^ = 0.065). The estimate was similar with a bootstrap 95% CI (0.15–3.73) and permutation testing (*p* = 0.029), after mutual adjustment for vegetable and fermented dairy intake (*β* = 2.04 pp, *p* = 0.026), among participants aged 18–25 years (*β* = 1.88 pp, *p* = 0.040), among women (*β* = 2.02 pp, *p* = 0.062), and among participants with ≥0.5 years of follow-up (*β* = 2.94 pp, *p* = 0.004). The association was weaker and not statistically significant in analyses without baseline adjustment (unadjusted change: *β* = 1.01 pp, *p* = 0.29; LMM: *β* = 0.76 pp/year, *p* = 0.20).

Most participants rated their subjective sleep quality as fairly good (score 1, *n* = 57) or fairly bad (score 2, *n* = 18); five rated it as very good (score 0); and none rated it as very bad (score 3). None of the other PSQI components was associated with follow-up BF% (all *p* ≥ 0.24; Supplementary Table S6).

### 3.5. Characteristics of Participants in the Extreme Quartiles of Body Fat Percentage Change

To further explore factors associated with substantial inter-individual differences in body fat change, participants were divided into quartiles according to the magnitude of BF% change, and the extreme quartiles were compared (Supplementary Table S7). The bottom quartile comprised participants with the greatest decreases in BF% (≤−2.45 pp), whereas the top quartile comprised those with the greatest increases (≥3.38 pp). Compared with participants in the bottom quartile, those in the top quartile, i.e., those who gained more body fat, were more likely to carry the *FTO* rs1421085 T allele (*p* = 0.02; FDR *q* = 0.157) (Supplementary Table S8), consumed fewer vegetables (*p* = 0.004; FDR *q* = 0.036), and had longer sleep durations (*p* = 0.028; FDR *q* = 0.126) (Supplementary Table S9). No significant differences between the extreme quartiles were observed for the other seven polymorphisms or the other behavioural factors examined. We also explored combinations of genetic and lifestyle factors in relation to the extreme quartiles of BF% change. Several nominal associations were observed (Supplementary Table S10), but none remained statistically significant after correction for multiple comparisons (all FDR *q* = 0.58). In Firth penalized logistic regression adjusted for baseline BF%, age, sex and follow-up duration, the differences in vegetable intake (*p* = 0.18) and sleep duration (*p* = 0.16) were not significant (Supplementary Table S9), and none of the combinations remained significant after FDR correction (smallest adjusted *q* = 0.58; Supplementary Table S10).

## 4. Discussion

To our knowledge, this is the first prospective study to examine the eight selected obesity-associated polymorphisms and lifestyle factors in relation to longitudinal changes in body fat percentage in young adults from Russia. In the primary analyses, none of the eight polymorphisms were associated with follow-up BF% after adjustment for baseline BF%, follow-up duration, age and sex, and none of the lifestyle exposures showed an FDR-significant association. Nevertheless, several exploratory findings warrant consideration. The data placed informative bounds on the magnitude of the effects of the studied genetic variants, worse subjective sleep quality was associated with higher follow-up BF%, and comparison of participants at the extremes of BF% change identified differences in vegetable intake, sleep duration and *FTO* genotype. These findings should be interpreted in the context of the modest sample size, incomplete genotyping and multiple exploratory analyses.

The absence of associations between the individual polymorphisms and BF% change should not be interpreted as evidence that these variants have no influence on adiposity trajectories. Individual adiposity loci generally have small effects on BMI and BF% [2,3], and the SNP-based heritability of BMI change is substantially lower than that of BMI [4]. Longitudinal studies that have detected genetic contributions to body-composition trajectories have generally included much larger samples with repeated measurements [5,6,7]. Our estimates nevertheless provide useful information for future research. For seven of the eight variants, the upper 95% confidence limits were compatible with per-risk-allele increases in follow-up BF% of no more than approximately 0.4–2.4 pp over the follow-up period, although the confidence interval for *PATJ* was wider and extended to 4.7 pp. The effect estimates and standard errors provided here may therefore contribute to future meta-analyses of longitudinal adiposity phenotypes.

The nominal association of the *TFAP2B* risk allele with a smaller increase in BF% in analyses without baseline adjustment is unlikely to represent a robust longitudinal genetic association. The same allele was associated with a non-significantly higher baseline BF%, and the association with BF% change disappeared after adjustment for baseline adiposity. Moreover, baseline BF% was inversely correlated with subsequent change in our cohort (*r* = −0.40), a pattern that can arise partly from mathematical coupling of baseline values with change scores and from regression to the mean [10]. This finding illustrates the importance of accounting for baseline adiposity when analyzing longitudinal changes in BF%, particularly in a small cohort with substantial inter-individual variation in baseline adiposity [11,12].

The extreme-quartile analysis provided additional insight into this heterogeneity. Participants in the upper quartile of BF% change, who had the greatest increases in body fat, consumed fewer vegetables than those in the lower quartile, and this difference remained significant after FDR correction (*q* = 0.036). This exploratory finding is consistent with previous evidence linking greater vegetable consumption with more favorable adiposity-related outcomes in young adults [23], although the present analysis cannot establish a causal relationship. However, the two extreme quartiles differed substantially in baseline BF% (19.1% vs. 26.4%), so unadjusted comparisons between them may partly reflect baseline adiposity and regression to the mean [10]; after adjustment for baseline BF%, age, sex and follow-up duration, the difference in vegetable intake was not significant. Vegetable intake was assessed using a single frequency item and showed limited variability, and the extreme-quartile comparison involved a relatively small number of participants. The finding should therefore be considered hypothesis-generating and evaluated using more detailed dietary assessment in larger longitudinal cohorts.

The same extreme-quartile analysis identified nominal differences in *FTO* rs1421085 allele carriage and sleep duration, with participants in the upper quartile more likely to carry the reported *FTO* allele and to report longer sleep duration. Neither finding survived correction for multiple comparisons (*q* = 0.157 and 0.126, respectively). These signals were not accompanied by significant associations in the primary continuous-outcome analysis. They may nevertheless indicate that factors associated with adiposity change are not necessarily detected consistently when the outcome is analyzed only as a continuous adjusted measure, particularly in a small sample. However, the extreme-quartile approach also reduces statistical power and can produce unstable estimates, so these findings require independent replication.

We also explored combinations of genetic and lifestyle factors in relation to the extreme quartiles of BF% change. Several nominal associations were observed, but none survived correction for multiple comparisons (all FDR *q* = 0.58). These results provide no robust evidence for specific combinations of the studied factors, but they illustrate the potential for interactions or clustering of risk-related characteristics to contribute to divergent adiposity trajectories. Larger studies with sufficient power for interaction analyses will be needed to distinguish reproducible combinations from chance findings.

Worse subjective sleep quality was also nominally associated with higher follow-up BF% in the primary ANCOVA model. Each one-point-higher PSQI subjective sleep-quality score was associated with a 1.96 pp higher follow-up BF%, and the estimate remained similar in several sensitivity analyses, including mutual adjustment for vegetable and fermented dairy intake and analyses restricted by age, sex or follow-up duration. However, the association did not survive correction for multiple testing (*q* = 0.11), was weaker and non-significant when baseline BF% was not included in the model, and was based predominantly on two response categories, with most participants reporting either fairly good or fairly bad sleep quality. Subjective sleep quality may capture aspects of sleep, including perceived restorative quality, that are not fully reflected by sleep duration or the PSQI global score. Poor sleep has been associated with altered appetite regulation, eating behaviour and circadian timing, and longitudinal studies have linked sleep disturbances with subsequent adiposity gain [8,24]. The present finding should therefore be regarded as a hypothesis for further investigation rather than evidence of an established longitudinal association.

Body-fat trajectories were markedly heterogeneous despite the small mean change. Mean BF% increased by less than 0.5 pp overall, yet 40% of participants gained at least 2 pp and 30% lost at least 2 pp, with substantial numbers showing changes of ≥5 pp in either direction. The extreme-quartile analysis further demonstrated that participants with the greatest increases and decreases differed in several behavioural and genetic characteristics, although most of these differences were not robust to multiple-testing correction. Thus, average change may conceal substantially divergent individual trajectories during young adulthood, a period characterised by changes in diet, sleep, physical activity and other lifestyle factors [1]. Some of the observed variability will also reflect measurement error inherent to BIA. Nevertheless, the strong correlation between baseline and follow-up BF% (*r* = 0.80) indicates substantial rank-order stability across the two assessments. Identifying predictors of divergent trajectories may therefore be a more informative objective for future studies than focusing solely on mean change.

We found no association of vegetable or fermented dairy intake with follow-up BF% in the primary ANCOVA analysis. The apparent difference in vegetable intake between the extreme quartiles suggests that the relationship may be more readily detected when individuals with the largest contrasting changes are considered, although this interpretation remains speculative. Previous evidence has linked vegetable intake with lower adiposity in young adults [24], whereas evidence concerning dairy and fermented dairy consumption is heterogeneous [25,26]. In our study, both dietary exposures were assessed using single, non-validated frequency items, with relatively limited variation in reported intake. These characteristics reduce the ability to detect dose–response relationships and may partly explain the absence of associations in the primary analysis.

Several strengths should be considered. This study used a prospective design with repeated BF% measurements obtained using the same device and under standardized measurement conditions, with exact follow-up dates available. The primary analyses accounted for baseline adiposity and follow-up duration and incorporated multiple approaches to assess the robustness of the genetic findings, including robust standard errors, bootstrap confidence intervals and permutation testing. The study also characterised the substantial inter-individual variation in BF% change and included an exploratory comparison of participants at the extremes of the distribution.

The limitations are considerable. The sample was small and predominantly female (82%), so the findings may not generalize to young men. Genotype data were incomplete, and the genotyped subsample differed from the non-genotyped participants in BF% change, which may introduce selection bias into the genetic analyses. BIA is sensitive to hydration status and other physiological and measurement conditions, and measurement error is particularly relevant when the average longitudinal change is small. Dietary exposures were assessed using single, non-validated frequency items, and lifestyle exposures were measured at a single time point. Physical activity, energy intake, alcohol consumption, smoking, socioeconomic factors and major life events were not assessed, leaving substantial potential for residual confounding. Follow-up duration varied between participants, although it was not correlated with BF% change and was included in the primary models. The extreme-quartile and combination analyses were exploratory and involved multiple comparisons, increasing the likelihood of chance findings despite FDR correction. Finally, baseline data from 80 of the 84 participants were included in our previous cross-sectional study [9]. The present analysis differs in design and outcome, focusing on longitudinal BF% change, and none of the longitudinal follow-up data have been reported previously.

## 5. Conclusions

In this prospective study of young adults from Russia, none of the eight selected obesity-associated polymorphisms were associated with longitudinal BF% change in the primary baseline-adjusted analyses. The estimates provide bounds on the magnitude of the genetic effects that could be detected in this sample and may be useful for future meta-analyses. Worse subjective sleep quality was nominally associated with higher follow-up BF%, while an exploratory comparison of participants with the greatest increases and decreases in BF% identified nominal differences in vegetable intake, *FTO* allele carriage and sleep duration. However, the quartiles differed substantially in baseline BF%, and none of these differences remained significant after FDR correction or, for vegetable intake and sleep duration, after adjustment for baseline BF%; therefore, findings require replication. Several combinations of genetic and lifestyle factors were also nominally associated with extreme BF% change, but none survived correction for multiple comparisons. Overall, the marked inter-individual variability in BF% trajectories despite a small average change highlight the need for larger longitudinal studies integrating genetic, dietary, sleep and other behavioural factors to identify reproducible predictors of adiposity change during young adulthood.

## Figures and Tables

**Table 1:** Characteristics of participants at baseline and follow-up.

Characteristic	All Participants (*n* = 84)	Men (*n* = 15)	Women (*n* = 69)
**Age, years**			
Mean ± SD	20.5 ± 2.6	20.1 ± 2.0	20.5 ± 2.7
Range	18.0 to 27.0	18.0 to 25.0	18.0 to 27.0
**Body fat at baseline, %**			
Mean ± SD	22.4 ± 7.0	15.3 ± 6.0	24.0 ± 6.3
Range	6.3 to 37.0	6.3 to 27.4	8.2 to 37.0
**Body fat at follow-up, %**			
Mean ± SD	22.9 ± 6.6	17.0 ± 6.0	24.2 ± 6.1
Range	8.9 to 39.3	9.1 to 28.6	8.9 to 39.3
**Change in body fat, percentage points**			
Mean ± SD	0.49 ± 4.36	1.61 ± 3.97	0.25 ± 4.43
Range	−10.10 to 11.10	−5.10 to 11.10	−10.10 to 10.90
Paired *t*-test for change, *p*	0.31	0.14	0.65

Change in body fat is calculated as follow-up minus baseline body fat percentage and is expressed in percentage points.

**Table 2:** Genotype distributions of the eight polymorphisms.

Polymorphism	Risk Allele	*n*	Genotype Counts	MAF	HWE *p*
*ADCY3* rs11676272	G	59	AA 20/AG 28/GG 11	0.42	0.80
*RERE* rs301806	T	47	CC 9/CT 21/TT 17	0.41	0.56
*FTO* rs1421085	C	58	TT 16/TC 30/CC 12	0.47	1.00
*TNRC6B* rs4821940	T	43	CC 15/CT 22/TT 6	0.40	0.76
*PATJ* rs12140153	G	36	TT 1/TG 4/GG 31	0.08	0.20
*TFAP2B* rs734597	A	50	GG 25/GA 20/AA 5	0.30	0.74
*MMS22L* rs9385223	G	57	TT 44/TG 13/GG 0	0.11	1.00
*SLC22A3* rs9364554	T	65	CC 38/CT 24/TT 3	0.23	1.00

Genotype counts are shown according to the number of risk alleles (0/1/2). MAF, minor allele frequency; HWE, exact test of Hardy–Weinberg equilibrium.

**Table 3:** Associations of genetic variants and lifestyle exposures with follow-up body fat percentage in the primary ANCOVA model.

Exposure	*n*	*β* (95% CI), pp	*p*	*q*	Partial *R*^2^
**Genetic variants (per risk allele)**					
*ADCY3* rs11676272	59	−0.30 (−1.69 to 1.09)	0.67	0.88	0.004
*RERE* rs301806	47	−1.11 (−2.63 to 0.41)	0.15	0.59	0.050
*FTO* rs1421085	58	−0.87 (−2.39 to 0.64)	0.25	0.67	0.025
*TNRC6B* rs4821940	43	0.45 (−1.52 to 2.42)	0.65	0.88	0.006
*PATJ* rs12140153	36	1.30 (−2.12 to 4.71)	0.44	0.88	0.020
*TFAP2B* rs734597	50	−1.34 (−3.05 to 0.36)	0.12	0.59	0.054
*MMS22L* rs9385223	57	−0.18 (−2.50 to 2.14)	0.88	0.88	0.000
*SLC22A3* rs9364554	65	−0.20 (−1.81 to 1.40)	0.80	0.88	0.001
**Lifestyle exposures (per unit)**					
Vegetable intake (1–6)	84	0.25 (−0.75 to 1.25)	0.63	0.83	0.003
Fermented dairy intake (1–6)	84	−0.02 (−0.67 to 0.63)	0.96	0.96	0.000
PSQI global score (0–21)	80	0.30 (−0.17 to 0.78)	0.21	0.42	0.021
PSQI subjective sleep quality (0–3)	80	1.96 (0.23 to 3.68)	0.027	0.11	0.065

Follow-up BF% was adjusted for baseline BF%, follow-up duration, age, and sex. *β*, difference in follow-up BF% (percentage points, pp) per risk allele or per unit of exposure; *q*, Benjamini–Hochberg false discovery rate within the genetic (eight tests) and lifestyle (four tests) families; PSQI, Pittsburgh Sleep Quality Index, with higher scores indicating poorer sleep quality.

## Data Availability

The data presented in this study are available on request from the corresponding author because of privacy and ethical restrictions.

## Supplementary Materials

The following supporting information can be downloaded at https://www.mdpi.com/article/10.3390/nu18213679/s1: File S1: Lifestyle questionnaire items used in the study (English translation); Table S1: Associations of the eight polymorphisms with body fat percentage in all models (summary statistics); Table S2: Genotype-specific values, ANCOVA with genotype as a categorical variable and within-genotype change in body fat percentage; Table S3: Linear mixed models of change in body fat percentage (not adjusted for baseline); Table S4: Associations of lifestyle exposures with body fat percentage in all models; Table S5: Primary ANCOVA model in subgroups; Table S6: PSQI global score and components and follow-up body fat percentage (primary ANCOVA model); Table S7: Characteristics of participants in the upper and lower quartiles of change in body fat percentage; Table S8: Genotype distributions and risk-allele frequencies of the eight polymorphisms in the upper and lower quartiles of change in body fat percentage; Table S9: Selected lifestyle variables in the upper and lower quartiles of change in body fat percentage; Table S10: Combinations of genetic and lifestyle factors in the upper and lower quartiles of change in body fat percentage.

## Abbreviations

The following abbreviations are used in this manuscript:

ANCOVA	analysis of covariance
BF%	body fat percentage
BF_01_	Bayes factor in favour of the null hypothesis
BIA	bioelectrical impedance analysis
BMI	body mass index
CI	confidence interval
FDR	false discovery rate
GWAS	genome-wide association study
HC3	heteroscedasticity-consistent standard errors, type 3
LMM	linear mixed model
MAF	minor allele frequency
pp	percentage points
PSQI	Pittsburgh Sleep Quality Index
SNP	single-nucleotide polymorphism
*ADCY3*	adenylate cyclase 3 gene
*FTO*	FTO alpha-ketoglutarate-dependent dioxygenase gene
*MMS22L*	MMS22-like, DNA repair protein gene
*PATJ*	PATJ crumbs cell polarity complex component gene
*RERE*	Arginine–glutamic acid dipeptide repeats gene
*SLC22A3*	solute carrier family 22-member 3 gene
*TFAP2B*	transcription factor AP-2 beta gene
*TNRC6B*	trinucleotide repeat-containing adaptor 6B gene
