# Supplementary material for: Genetic Variants, Lifestyle Factors and Changes in Body Fat Percentage in Young Adults: An Exploratory Prospective Study
Source: Nutrients. 2026 Nov 6;18(21):3679. doi: 10.3390/nu18213679 (PMC13766046; doi:10.3390/nu18213679)
Supplement: Supplementary file 1 [file nutrients-18-03679-s001.zip › nutrients-4567320-supplementary.pdf]

Supplementary Materials

Genetic Variants, Lifestyle Factors and Changes in Body Fat Percentage in Young Adults: An Exploratory Prospective Study

Egorova E.S., Valeeva E.V., Ahmetov I.I.

Abbreviations: BF%, body fat percentage; pp, percentage points; CI, confidence interval; FDR, false discovery rate (Benjamini–Hochberg); *q*, FDR-adjusted *p*; ANCOVA, analysis of covariance; LMM, linear mixed model; PSQI, Pittsburgh Sleep Quality Index. Genotypes are coded as the number of risk alleles (additive model) unless stated otherwise.

File S1. Lifestyle questionnaire items used in the study (English translation)

The questionnaires were administered in Russian. Only the items analysed in the present study are shown. The English translation of the dietary item was prepared by the authors; the original Russian wording is given for reference.

A. Dietary intake

Question. *Do you eat the following foods?* (Russian original: «Употребляете ли Вы в пищу:»)

Please mark one answer for each food group.

Food group	Never (1)	Less than once a month (2)	1-2 times a month (3)	1-2 times a week (4)	3-4 times a week (5)	Daily (6)
(a) Vegetables («Овощи»)	☐	☐	☐	☐	☐	☐
(b) Fermented dairy products («Молочно-кислые продукты»)	☐	☐	☐	☐	☐	☐

Scoring. Responses were coded 1 (never) to 6 (daily) and analysed as ordinal scores; higher scores indicate more frequent consumption. Each food group was analysed separately.

B. Pittsburgh Sleep Quality Index (PSQI)

Sleep quality over the previous month was assessed with the Pittsburgh Sleep Quality Index [14], administered as the Russian translation by E.A. Semenova and K.V. Danilenko [15]. The PSQI is a copyrighted instrument (© University of Pittsburgh) and is therefore not reproduced here; the original English version is available from its developers. The self-rated items and the scoring of the seven components are summarised below.

Table 1. PSQI items (content summarised).

Item	Content	Response format
1	Usual bedtime	Clock time
2	Usual time to fall asleep (sleep latency)	Minutes
3	Usual getting-up time	Clock time
4	Hours of actual sleep per night	Hours
5a–5j	How often trouble sleeping because of: (a) not falling asleep within 30 min; (b) waking in the middle of the night or early morning; (c) getting up to use the bathroom; (d) not breathing comfortably; (e) coughing or snoring loudly; (f) feeling too cold; (g) feeling too hot; (h) bad dreams; (i) pain; (j) other reason(s)	0 = not during the past month; 1 = less than once a week; 2 = once or twice a week; 3 = three or more times a week
6	Overall rating of sleep quality	0 = very good; 1 = fairly good; 2 = fairly bad; 3 = very bad
7	Use of medicine to help sleep	Frequency, 0–3 (as for item 5)
8	Trouble staying awake while driving, eating or during social activity	Frequency, 0–3 (as for item 5)
9	Problem keeping up enthusiasm to get things done	0 = no problem at all to 3 = a very big problem

Table 2. Scoring of PSQI components.

Component	Items used	Scoring (0–3)
C1. Subjective sleep quality †	6	Item 6 score
C2. Sleep latency	2, 5a	Item 2: ≤15 min = 0, 16–30 = 1, 31–60 = 2, >60 = 3; added to item 5a; sum 0 = 0, 1–2 = 1, 3–4 = 2, 5–6 = 3
C3. Sleep duration	4	>7 h = 0; 6–7 h = 1; 5–6 h = 2; <5 h = 3
C4. Habitual sleep efficiency	1, 3, 4	Hours asleep / hours in bed × 100: ≥85% = 0; 75–84% = 1; 65–74% = 2; <65% = 3
C5. Sleep disturbances	5b–5j	Sum 0 = 0; 1–9 = 1; 10–18 = 2; 19–27 = 3

C6. Use of sleeping medication	7	Item 7 score
C7. Daytime dysfunction	8, 9	Sum 0 = 0; 1–2 = 1; 3–4 = 2; 5–6 = 3
**Global PSQI score**	C1–C7	**Sum of components, 0–21**; higher scores indicate worse sleep quality; >5 indicates poor sleep

† Component 1 (subjective sleep quality) was analysed as a secondary exploratory exposure; the global score was the prespecified sleep exposure.

Supplementary Tables

Table S1. Associations of the eight polymorphisms with body fat percentage in all models (summary statistics).

Polymorphism	*n*	*β* per risk allele, pp	SE	95% CI	*p*	*q*
**Baseline BF% (cross-sectional)**						
ADCY3 rs11676272	59	1.66	1.13	−0.61 to 3.93	0.15	0.59
RERE rs301806	47	0.98	1.24	−1.53 to 3.48	0.44	0.70
FTO rs1421085	58	0.51	1.33	−2.15 to 3.16	0.70	0.70
TNRC6B rs4821940	43	−0.75	1.62	−4.02 to 2.52	0.65	0.70
PATJ rs12140153	36	−2.39	2.36	−7.20 to 2.43	0.32	0.64
TFAP2B rs734597	50	2.03	1.37	−0.73 to 4.78	0.15	0.59
MMS22L rs9385223	57	2.25	1.98	−1.72 to 6.22	0.26	0.64
SLC22A3 rs9364554	65	0.83	1.35	−1.87 to 3.52	0.54	0.70
**Primary: ANCOVA, follow-up BF% adj. baseline, follow-up, age, sex**						
ADCY3 rs11676272	59	−0.30	0.69	−1.69 to 1.09	0.67	0.88
RERE rs301806	47	−1.11	0.75	−2.63 to 0.41	0.15	0.59
FTO rs1421085	58	−0.87	0.76	−2.39 to 0.64	0.25	0.67
TNRC6B rs4821940	43	0.45	0.97	−1.52 to 2.42	0.65	0.88
PATJ rs12140153	36	1.30	1.67	−2.12 to 4.71	0.44	0.88
TFAP2B rs734597	50	−1.34	0.85	−3.05 to 0.36	0.12	0.59
MMS22L rs9385223	57	−0.18	1.15	−2.50 to 2.14	0.88	0.88
SLC22A3 rs9364554	65	−0.20	0.80	−1.81 to 1.40	0.80	0.88
**ANCOVA without follow-up time**						
ADCY3 rs11676272	59	−0.32	0.69	−1.69 to 1.06	0.65	0.86
RERE rs301806	47	−1.11	0.74	−2.61 to 0.38	0.14	0.56
FTO rs1421085	58	−0.80	0.74	−2.29 to 0.70	0.29	0.77
TNRC6B rs4821940	43	0.47	0.95	−1.45 to 2.38	0.62	0.86
PATJ rs12140153	36	1.23	1.66	−2.15 to 4.61	0.46	0.86
TFAP2B rs734597	50	−1.34	0.83	−3.01 to 0.33	0.11	0.56
MMS22L rs9385223	57	−0.17	1.15	−2.47 to 2.13	0.88	0.88
SLC22A3 rs9364554	65	−0.20	0.80	−1.80 to 1.39	0.80	0.88
**Change in BF%, not adjusted for baseline**						
ADCY3 rs11676272	59	−0.79	0.74	−2.28 to 0.69	0.29	0.48
RERE rs301806	47	−1.45	0.84	−3.14 to 0.25	0.09	0.37
FTO rs1421085	58	−0.94	0.83	−2.61 to 0.72	0.26	0.48
TNRC6B rs4821940	43	0.69	1.04	−1.42 to 2.80	0.51	0.59
PATJ rs12140153	36	1.78	1.70	−1.67 to 5.24	0.30	0.48
TFAP2B rs734597	50	−1.89	0.89	−3.67 to −0.11	0.04	0.31
MMS22L rs9385223	57	−0.87	1.28	−3.43 to 1.69	0.50	0.59

Polymorphism	*n*	*β* per risk allele, pp	SE	95% CI	*p*	*q*
SLC22A3 rs9364554	65	−0.45	0.88	−2.21 to 1.32	0.62	0.62
**Annualised change (pp/year), follow-up ≥0.5 years**						
ADCY3 rs11676272	51	−0.37	0.50	−1.38 to 0.64	0.47	0.61
RERE rs301806	41	−1.26	0.63	−2.53 to 0.01	0.05	0.23
FTO rs1421085	50	−0.57	0.58	−1.74 to 0.59	0.33	0.61
TNRC6B rs4821940	35	0.47	0.75	−1.06 to 2.01	0.54	0.61
PATJ rs12140153	29	0.48	1.21	−2.01 to 2.96	0.70	0.70
TFAP2B rs734597	42	−1.19	0.61	−2.42 to 0.04	0.06	0.23
MMS22L rs9385223	55	−0.66	0.86	−2.38 to 1.06	0.44	0.61
SLC22A3 rs9364554	56	−0.44	0.63	−1.71 to 0.84	0.49	0.61

Baseline model adjusted for age and sex; ANCOVA models additionally for baseline BF% (and follow-up duration where stated); change models adjusted for age and sex. *q*: FDR over the eight polymorphisms within each model. β and SE are provided as summary statistics for meta-analysis.

Table S2. Genotype-specific values, ANCOVA with genotype as a categorical variable and within-genotype change in body fat percentage.

Polymorphism	Grouping	*n*	*F* (df)	*p*	*q*	Partial η^2	Slopes *p*	Shapiro–Wilk *p*	Levene *p*
ADCY3 rs11676272	3 genotypes	59	0.39 (2, 52)	0.68	0.88	0.015	0.36	0.79	0.75
RERE rs301806	3 genotypes	47	1.21 (2, 40)	0.31	0.66	0.057	0.72	0.75	0.88
FTO rs1421085	3 genotypes	58	0.90 (2, 51)	0.41	0.66	0.034	0.15	0.57	0.98
TNRC6B rs4821940	3 genotypes	43	0.99 (2, 36)	0.38	0.66	0.052	0.02	0.96	0.51
PATJ rs12140153	recessive (rare protective homozygotes merged)	36	0.68 (1, 30)	0.41	0.66	0.022	0.09	0.98	0.17
TFAP2B rs734597	3 genotypes	50	2.46 (2, 43)	0.10	0.66	0.103	0.31	0.93	0.75
MMS22L rs9385223	3 genotypes	57	0.02 (1, 51)	0.88	0.88	0.000	0.48	0.38	0.18
SLC22A3 rs9364554	dominant (rare risk homozygotes merged)	65	0.03 (1, 59)	0.85	0.88	0.001	0.55	0.98	0.43

Part A. ANCOVA of follow-up BF% with genotype as a categorical factor, adjusted for baseline BF%, follow-up duration, age and sex. Homozygous groups with <5 participants were merged with heterozygotes. Slopes *p*: test of homogeneity of regression slopes (genotype × baseline BF%).

Polymorphism	Genotype	*n*	Baseline BF%	Follow-up BF%	Change (95% CI), pp	Paired *p*	*q*
ADCY3 rs11676272	AA	20	21.1	23.0	1.89 (−0.20 to 3.99)	0.07	0.23
ADCY3 rs11676272	AG	28	22.7	24.5	1.84 (0.46 to 3.22)	0.01	0.09
ADCY3 rs11676272	GG	11	25.0	25.0	−0.01 (−2.90 to 2.88)	0.99	0.99
RERE rs301806	CC	9	21.6	24.8	3.20 (0.82 to 5.58)	0.01	0.10
RERE rs301806	CT	21	24.1	25.4	1.31 (−0.64 to 3.26)	0.18	0.34
RERE rs301806	TT	17	23.5	23.8	0.29 (−1.99 to 2.58)	0.79	0.93
FTO rs1421085	TT	16	19.5	22.1	2.62 (0.72 to 4.53)	0.01	0.09
FTO rs1421085	TC	30	23.8	25.5	1.64 (0.28 to 2.99)	0.02	0.10
FTO rs1421085	CC	12	23.5	23.8	0.25 (−3.03 to 3.53)	0.87	0.93
TNRC6B rs4821940	CC	15	23.0	23.5	0.50 (−0.98 to 1.98)	0.48	0.67
TNRC6B rs4821940	CT	22	23.7	25.5	1.79 (−0.52 to 4.10)	0.12	0.29
TNRC6B rs4821940	TT	6	23.8	24.6	0.80 (−3.06 to 4.66)	0.62	0.82
PATJ rs12140153	TT	1	30.8	28.7	−2.10	—	—
PATJ rs12140153	TG	4	22.6	23.5	0.90 (−10.80 to 12.60)	0.82	0.93
PATJ rs12140153	GG	31	21.7	24.0	2.35 (0.92 to 3.78)	0.002	0.08
TFAP2B rs734597	GG	25	21.5	23.7	2.20 (0.58 to 3.82)	0.010	0.09
TFAP2B rs734597	GA	20	24.2	25.6	1.41 (−0.29 to 3.12)	0.10	0.28
TFAP2B rs734597	AA	5	26.4	23.4	−3.08 (−9.42 to 3.26)	0.25	0.40
MMS22L rs9385223	TT	44	22.2	23.8	1.60 (0.32 to 2.88)	0.02	0.10
MMS22L rs9385223	TG	13	24.1	24.9	0.80 (−1.13 to 2.73)	0.38	0.57

Polymorphism	Genotype	*n*	Baseline BF%	Follow-up BF%	Change (95% CI), pp	Paired *p*	*q*
MMS22L rs9385223	GG	0	—	—	—	—	—
SLC22A3 rs9364554	CC	38	21.9	23.5	1.53 (0.10 to 2.96)	0.04	0.13
SLC22A3 rs9364554	CT	24	23.0	24.2	1.18 (−0.38 to 2.74)	0.13	0.29
SLC22A3 rs9364554	TT	3	26.9	26.6	−0.33 (−9.06 to 8.39)	0.88	0.93

Part B. Mean BF% and within-genotype change (whole cohort). Paired *t*-tests; *q*: FDR across all 77 within-genotype tests (whole cohort and subgroups). Comparison of change between genotypes (one-way ANOVA): *ADCY3* $p = 0.39$; *RERE* $p = 0.25$; *FTO* $p = 0.30$; *TNRC6B* $p = 0.65$; *PATJ* $p = 0.53$; *TFAP2B* $p = 0.03$; *MMS22L* $p = 0.53$; and *SLC22A3* $p = 0.74$. Significant within-group change reflects the overall gain in the genotyped subsample and group size, not genotype; a difference in significance between groups is not evidence of a difference between them.

Table S3. Linear mixed models of change in body fat percentage (not adjusted for baseline).

Exposure	n	β time × exposure (95% CI), pp/year	p	q	β exposure at baseline, pp	ICC
ADCY3 rs11676272	59	−0.39 (−1.20 to 0.42)	0.33	0.53	1.59	0.77
RERE rs301806	47	−0.81 (−1.76 to 0.14)	0.09	0.25	0.85	0.74
FTO rs1421085	58	−0.75 (−1.73 to 0.23)	0.13	0.26	0.57	0.79
TNRC6B rs4821940	43	0.12 (−0.92 to 1.17)	0.81	0.81	−0.66	0.77
PATJ rs12140153	36	1.52 (−0.28 to 3.32)	0.10	0.25	−2.80	0.76
TFAP2B rs734597	50	−1.04 (−1.98 to −0.11)	0.03	0.24	2.02	0.78
MMS22L rs9385223	57	−0.38 (−1.70 to 0.94)	0.56	0.75	2.22	0.77
SLC22A3 rs9364554	65	−0.20 (−1.18 to 0.79)	0.69	0.79	0.80	0.77
Vegetable intake (1–6)	84	0.11 (−0.54 to 0.77)	0.73	0.73	0.51	0.75
Fermented dairy intake (1–6)	84	0.28 (−0.13 to 0.69)	0.18	0.30	−0.60	0.75
PSQI global score (0–21)	80	0.19 (−0.12 to 0.51)	0.22	0.30	0.19	0.77
PSQI subjective sleep quality (0–3)	80	0.76 (−0.41 to 1.93)	0.20	0.30	3.08	0.75

Random-intercept model fitted by restricted maximum likelihood to both measurements; time in years; adjusted for age, sex and their interactions with time. β time × exposure: difference in annual change in BF% per risk allele or per unit of exposure. *q*: FDR within the genetic (8) and lifestyle (4) families. Annualised change models are shown in Tables S1 and S4.

Table S4. Associations of lifestyle exposures with body fat percentage in all models.

Exposure	*n*	β per unit (95% CI), pp	*p*	*q*	Partial R^2
**Primary: ANCOVA, follow-up BF% adj. baseline, follow-up, age, sex**					
Vegetable intake (1–6)	84	0.25 (−0.75 to 1.25)	0.63	0.83	0.003
Fermented dairy intake (1–6)	84	−0.02 (−0.67 to 0.63)	0.96	0.96	0.000
PSQI global score (0–21)	80	0.30 (−0.17 to 0.78)	0.21	0.42	0.021
PSQI subjective sleep quality (0–3)	80	1.96 (0.23 to 3.68)	0.03	0.11	0.065
**ANCOVA without follow-up time**					
Vegetable intake (1–6)	84	0.26 (−0.75 to 1.27)	0.61	0.81	0.003
Fermented dairy intake (1–6)	84	0.05 (−0.60 to 0.70)	0.88	0.88	0.000
PSQI global score (0–21)	80	0.31 (−0.17 to 0.79)	0.20	0.41	0.021
PSQI subjective sleep quality (0–3)	80	1.98 (0.24 to 3.72)	0.03	0.10	0.064
**Change in BF%, not adjusted for baseline**					
Vegetable intake (1–6)	84	0.10 (−1.00 to 1.19)	0.86	0.86	0.000
Fermented dairy intake (1–6)	84	0.18 (−0.53 to 0.88)	0.62	0.82	0.003
PSQI global score (0–21)	80	0.25 (−0.27 to 0.77)	0.34	0.69	0.012
PSQI subjective sleep quality (0–3)	80	1.01 (−0.86 to 2.89)	0.29	0.69	0.015
**Annualised change (pp/year), follow-up ≥0.5 years**					
Vegetable intake (1–6)	73	−0.06 (−0.93 to 0.81)	0.89	1.00	0.000
Fermented dairy intake (1–6)	73	−0.00 (−0.57 to 0.57)	1.00	1.00	0.000
PSQI global score (0–21)	69	0.21 (−0.24 to 0.67)	0.35	0.70	0.014
PSQI subjective sleep quality (0–3)	69	0.83 (−0.84 to 2.50)	0.32	0.70	0.015

q: FDR over the four lifestyle exposures within each model.

Table S5. Primary ANCOVA model in subgroups.

Exposure	*n*	*β* (95% CI), pp	*p*
**Women only (n = 69; not adjusted for sex)**			
ADCY3 rs11676272	50	−0.57 (−2.04 to 0.89)	0.44
RERE rs301806	40	−1.29 (−2.86 to 0.27)	0.10
FTO rs1421085	49	−0.54 (−2.14 to 1.05)	0.50
TNRC6B rs4821940	38	−0.02 (−1.94 to 1.90)	0.98
PATJ rs12140153	31	1.10 (−2.29 to 4.48)	0.51
TFAP2B rs734597	43	−1.21 (−2.96 to 0.54)	0.17
MMS22L rs9385223	50	0.24 (−2.24 to 2.71)	0.85
SLC22A3 rs9364554	56	−0.05 (−1.71 to 1.61)	0.95
Vegetable intake (1–6)	69	0.03 (−1.09 to 1.15)	0.96
Fermented dairy intake (1–6)	69	−0.08 (−0.81 to 0.65)	0.83
PSQI global score (0–21)	65	0.26 (−0.27 to 0.79)	0.33
PSQI subjective sleep quality (0–3)	65	2.02 (−0.10 to 4.15)	0.06
**Participants aged 18–25 years (n = 81)**			
ADCY3 rs11676272	58	−0.30 (−1.70 to 1.10)	0.67
RERE rs301806	46	−1.09 (−2.66 to 0.48)	0.17
FTO rs1421085	56	−0.81 (−2.37 to 0.76)	0.31
TNRC6B rs4821940	42	0.72 (−1.36 to 2.79)	0.49
PATJ rs12140153	35	1.31 (−2.15 to 4.76)	0.45
TFAP2B rs734597	49	−1.38 (−3.11 to 0.35)	0.11
MMS22L rs9385223	54	−0.21 (−2.64 to 2.22)	0.86
SLC22A3 rs9364554	62	−0.22 (−1.89 to 1.45)	0.79
Vegetable intake (1–6)	81	0.35 (−0.67 to 1.38)	0.49
Fermented dairy intake (1–6)	81	−0.05 (−0.71 to 0.62)	0.89
PSQI global score (0–21)	77	0.29 (−0.20 to 0.77)	0.24
PSQI subjective sleep quality (0–3)	77	1.88 (0.09 to 3.66)	0.04
**Participants with follow-up ≥0.5 years (n = 73)**			
ADCY3 rs11676272	51	−0.07 (−1.53 to 1.39)	0.93
RERE rs301806	41	−1.59 (−3.39 to 0.22)	0.08
FTO rs1421085	50	−1.11 (−2.66 to 0.44)	0.16
TNRC6B rs4821940	35	−0.11 (−2.27 to 2.05)	0.92
PATJ rs12140153	29	0.87 (−2.81 to 4.54)	0.63
TFAP2B rs734597	42	−1.59 (−3.38 to 0.21)	0.08

**Exposure**	***n***	***β* (95% CI), pp**	***p***
MMS22L rs9385223	55	−0.59 (−2.97 to 1.79)	0.62
SLC22A3 rs9364554	56	−0.37 (−2.12 to 1.37)	0.67
Vegetable intake (1–6)	73	0.20 (−0.83 to 1.24)	0.69
Fermented dairy intake (1–6)	73	0.01 (−0.68 to 0.70)	0.98
PSQI global score (0–21)	69	0.56 (0.04 to 1.08)	0.04
PSQI subjective sleep quality (0–3)	69	2.94 (1.00 to 4.88)	0.004

Follow-up BF% adjusted for baseline BF%, follow-up duration, age and sex (sex omitted in women).

Table S6. PSQI global score and components and follow-up body fat percentage (primary ANCOVA model).

PSQI measure	*n*	*β* per point (95% CI), pp	*p*	*q* (8 PSQI tests)
Global score (0–21)	80	0.30 (−0.17 to 0.78)	0.21	0.66
C1 Subjective sleep quality	80	1.96 (0.23 to 3.68)	0.03	0.21
C2 Sleep latency	80	0.26 (−0.88 to 1.41)	0.65	0.96
C3 Sleep duration	80	−0.10 (−1.12 to 0.92)	0.85	0.96
C4 Habitual sleep efficiency	80	−1.71 (−9.81 to 6.39)	0.68	0.96
C5 Sleep disturbances	80	−0.05 (−1.93 to 1.83)	0.96	0.96
C6 Use of sleep medication	80	0.47 (−2.32 to 3.27)	0.74	0.96
C7 Daytime dysfunction	80	0.58 (−0.42 to 1.59)	0.25	0.66

Components scored 0–3 (higher = worse). Follow-up BF% adjusted for baseline BF%, follow-up duration, age and sex. *q*: FDR across the global score and seven components.

Table S7. Characteristics of participants in the upper and lower quartiles of change in body fat percentage.

Characteristic	Upper quartile (gain ≥3.38 pp)	Lower quartile (change ≤−2.45 pp)	*p*
n	21	21	
Women, *n* (%)	17 (81%)	19 (90%)	0.66
Age, years	21.0 ± 3.5	19.9 ± 1.9	0.22
Baseline BF%	19.1 ± 6.7	26.0 ± 5.7	<0.001
Follow-up BF%	24.9 ± 6.3	21.0 ± 5.7	0.04
Change in BF%, pp	+5.80 ± 2.35	−5.03 ± 2.37	<0.001
Follow-up, years	1.59 ± 0.64	1.63 ± 0.63	0.83

Quartiles of change in BF% (follow-up minus baseline) among 84 participants; the two middle quartiles are not shown. Values are *n*, *n* (%) or mean ± SD. *p:* Mann–Whitney test.

Table S8. Genotype distributions and risk-allele frequencies of the eight polymorphisms in the upper and lower quartiles of change in body fat percentage.

**Polymorphism**	**Risk allele**	***n*, upper quartile**	***n*, lower quartile**	**RAF, upper**	**RAF, lower**	***p***	***q***
ADCY3 rs11676272	G	18	9	0.333	0.556	0.201	0.362
RERE rs301806	T	12	9	0.458	0.778	0.066	0.264
FTO rs1421085	C	19	8	0.342	0.688	0.020	0.157
TNRC6B rs4821940	T	11	8	0.455	0.438	0.920	0.920
PATJ rs12140153	G	12	4	0.917	0.750	0.226	0.362
TFAP2B rs734597	A	14	9	0.214	0.500	0.104	0.278
MMS22L rs9385223	G	17	10	0.088	0.100	0.911	0.920
SLC22A3 rs9364554	T	19	11	0.237	0.227	0.862	0.920

Numbers are participants with genotype data in each quartile. RAF, risk-allele frequency. *p*: Mann–Whitney test, *q*: Benjamini–Hochberg FDR across the eight polymorphisms.

Table S9. Selected lifestyle variables in the upper and lower quartiles of change in body fat percentage.

Variable	*n*, upper/lower	Mean ± SD, upper quartile	Mean ± SD, lower quartile	*p*	*q*	Adjusted *p*
Vegetable intake (1–6)	21/19	4.00 ± 1.03	5.44 ± 0.86	0.004	0.036	0.181
Sleep duration	21/19	6.85 ± 0.92	6.04 ± 1.05	0.028	0.126	0.157
Fermented dairy intake (1–6)	21/19	5.15 ± 1.23	4.92 ± 1.44	0.645	1.000	0.246

Upper quartile, largest gain in BF%; lower quartile, largest loss. Data from the extended lifestyle questionnaire available for a subset of participants. *p*: Mann–Whitney test, *q*: Benjamini–Hochberg FDR within the lifestyle-related category (nine tests: vegetable consumption, fermented dairy product consumption, and seven items from the PSQI – excluding item C6, the use of sleep medication). Adjusted *p*: Firth penalized logistic regression adjusted for baseline BF%, age, sex and follow-up duration (penalized likelihood-ratio test).

Table S10. Combinations of genetic and lifestyle factors in the upper and lower quartiles of change in body fat percentage.

**Combination**	***n*, upper/lower**	**Upper quartile among carriers**	**Upper quartile among non-carriers**	***p***	***q***
RERE + *FTO*	12/8	33%	100%	0.013	0.58
ADCY3 + *FTO* + PSQI >5	18/7	33%	84%	0.018	0.58
FTO + *PATJ* + *TFAP2B*	12/3	50%	100%	0.044	0.58

Gene names denote carriage of ≥1 risk allele; PSQI >5, PSQI global score >5. *p*: two-sided Fisher's exact test, *q*: Benjamini–Hochberg FDR across all 74 combinations tested; only combinations with nominal $p < 0.05$ are shown.
